# The utility of the Brief Edinburgh Depression Scale (BEDS) in assessing severity of depression in advanced cancer patients

**DOI:** 10.1192/bjo.2021.161

**Published:** 2021-06-18

**Authors:** Zeryab Meyer, Christopher Shiels, Christopher Dowrick, Mari Lloyd-Williams

**Affiliations:** 1 School of Medicine, University of Liverpool; 2 Research Associate, Institute of Population Health Sciences, University of Liverpool; 3 Professor of Primary Medical Care, Institute of Population Health Sciences, University of Liverpool; 4 Professor and Honorary Consultant in Palliative Medicine, Academic Palliative and Supportive Care Studies Group (APSCSG), University of Liverpool

## Abstract

**Aims:**

When using an assessment tool, brevity and validity are essential. Although brief depression inventories exist, they rely heavily on the inclusion of somatic symptoms. This can be problematic in advanced cancer populations; weight loss and sleep disturbance are for the most part ubiquitous in these patients and may not necessarily be indicative of depression.

The Brief Edinburgh Depression Scale (BEDS) is a 6-item shortened version of the Edinburgh Depression Scale which has been validated for use in patients with advanced cancer and is used internationally. The BEDS cut off threshold of 6/18 indicates that depression may be present. However, the BEDS currently provides no information regarding severity. The aim of this study is to establish severity thresholds for the BEDS by comparing it to another depression scale: the commonly used, rigorously validated, Patient Health Questionnaire (PHQ-9).

**Method:**

284 advanced cancer patients attending hospice day services in the North West of England completed both the PHQ-9 and the BEDS. Mean participant age was 66.7 (Standard Deviation = 13.2) and the sample contained both males (n = 102, 36%) and females (n = 182, 64%). BEDS severity thresholds with the highest Sensitivity (Sn) and Specificity (Sp) were selected based on their ability to predict PHQ-9 categories.

**Result:**

A BEDS score of 4 to 6 was selected to indicate ‘mild depression’ (Sn = 81.7, Sp = 65); 7 to 8 ‘moderate depression’ (Sn = 74.8, Sp = 78.7); 9 to 11 ‘moderately severe depression’ (Sn = 82, Sp = 82.9) and 12 or more ‘severe depression’ (Sn = 63.2, Sp = 92.8). A linearly weighted kappa (with s weighting) showed a moderate level of agreement (0.47, 95% Confidence Interval: 0.40-0.54).

**Conclusion:**

The BEDS is a simple and brief tool used to screen for depression in advanced cancer patients. It is administered throughout the UK and multiple translation studies have enabled its global a (including in resource poor countries). The severity thresholds calculated here are derived from a large sample of patients with advanced cancer attending hospice services and demonstrate acceptable sensitivity and specificity in relation to the PHQ-9, a thoroughly validated reference standard. We conclude that the generated BEDS thresholds support use of the BEDS in determining the presence and severity of depression in advanced cancer populations.

